# Vertical Distribution of Epibenthic Freshwater Cyanobacterial *Synechococcus* spp. Strains Depends on Their Ability for Photoprotection

**DOI:** 10.1371/journal.pone.0020134

**Published:** 2011-05-18

**Authors:** Jens N. Lohscheider, Martina Strittmatter, Hendrik Küpper, Iwona Adamska

**Affiliations:** 1 Department of Biology, University of Konstanz, Konstanz, Germany; 2 Faculty of Biological Sciences and Institute of Physical Biology, University of South Bohemia, České Budejovice, Czech Republic; George Mason University, United States of America

## Abstract

**Background:**

Epibenthic cyanobacteria often grow in environments where the fluctuation of light intensity and quality is extreme and frequent. Different strategies have been developed to cope with this problem depending on the distribution of cyanobacteria in the water column.

**Principal Findings:**

Here we provide an experimental proof that the light intensity plays an important role in the vertical distribution of seven, closely related, epibenthic *Synechococcus* spp. strains isolated from various water depths from the littoral zone of Lake Constance in Germany and cultivated under laboratory conditions. Pigment analysis revealed that the amount of chlorophyll *a* and total carotenoids decreased with the time of light stress exposure in three phycoerythrin-rich strains collected from 7.0 m water depth and remained low during the recovery phase. In contrast, a constant level of chlorophyll *a* and either constant or enhanced levels of carotenoids were assayed in phycocyanin-rich strains collected from 1.0 and 0.5 m water depths. Protein analysis revealed that while the amount of biliproteins remained constant in all strains during light stress and recovery, the amount of D1 protein from photosystem II reaction centre was strongly reduced under light stress conditions in strains from 7.0 m and 1.0 m water depth, but not in strains collected from 0.5 m depth.

**Conclusion:**

Based on these data we propose that light intensity, in addition to light quality, is an important selective force in the vertical distribution of *Synechococcus* spp. strains, depending on their genetically fixed mechanisms for photoprotection.

## Introduction

Cyanobacteria are photosynthetically active prokaryotes, which share a high degree of similarity in structure and function of their photosynthetic apparatus with higher plants, green and red algae. Nevertheless, they differ in the structure of their light-harvesting antenna complexes. Unlike higher plants, most of the cyanobacteria lack chlorophyll (Chl) *a/b*-binding antenna proteins associated with photosystem II (PSII). Instead they contain high molecular mass multi-protein complexes, called phycobilisomes (PBS) that are attached to the cytosolic side of thylakoid membranes [1]. PBS are composed of various chromophore-binding phycobiliproteins and pigmented or non-pigmented protein linkers. Each PBS consists of phycobiliproteins, such as allophycocyanin (APC) building a core and phycocyanin (PC) and phycoerythrin (PE) building rods. Phycobiliproteins bind either blue phycocyanobilin or red phycoerythrobilin pigments [2].

Due to their maximal absorption in the green and orange regions of the light spectrum, their high abundance and a very efficient energy transfer of more than 95% [3], PBS allow cyanobacteria to grow in extreme habitats that are not available to other photosynthetically active organisms, e.g. in deep water, where light intensity is very low and light quality is restricted to green and blue regions of the light spectrum. This apparent advantage might quickly turn into a great disadvantage when sessile cyanobacterial cells are exposed to fluctuations of the water level, and therefore, to increased light intensities. Excess light might lead to the inhibition of photosynthetic functions and this process is referred to as photoinhibition [4]. At ambient temperatures, photoinhibition occurs primarily at the level of PSII and involves reversible inactivation of PSII due to the arrest of electron transport within this complex followed by irreversible photodamage to the subunits of PSII reaction centre by reactive oxygen species, where D1 protein is the main target of such damage [4]–[8]. Cyanobacteria developed several short- and long-term protective mechanisms to reduce or prevent photooxidative damage, like the exchange of photodamaged D1 protein of the PSII reaction centre [5]–[8], the accumulation of photoprotective carotenoids [9], or differential coupling of phycobiliproteins [10].

The D1 protein is the central component of the photosynthetic electron transport chain in the PSII reaction centre of cyanobacteria, algae and higher plants. It binds almost all cofactors necessary for electron transfer within PSII and is, therefore, extremely susceptible to photooxidative damage [4]–[8]. Rapid degradation of the photodamaged D1 protein and its replacement by a newly synthesised functional copy represent an important repair mechanism essential for cell survival under light stress conditions [4]–[8].

Carotenoids have been shown to be very important for photoprotection in higher plants and algae, where they absorb excess energy and dissipate it as heat, thereby limiting the formation of potentially destructive reactive oxygen species that can ultimately cause cell death [11], [12]. Such thermal energy dissipation can be mediated via the xanthophyll cycle pigment zeaxanthin [13], [14] or lutein [15], [16]. Although cyanobacteria do not possess a xanthophyll cycle, they are able to induce synthesis of the protective carotenoid zeaxanthin upon light stress [17], [18].

Some cyanobacteria are able to adapt to fluctuations in light quality by a change of the PC to PE ratio, in the process called complementary chromatic adaptation (CCA) [19], [20]. This ability for CCA in cyanobacteria allows maximal photosynthetic performance under light conditions unfavourable for other photosynthetic organisms. Light acclimation of at least some cyanobacteria also involves, as a short-term strategy, changes in the coupling of individual phycobiliproteins to the photosynthetic reaction centres [10].

It has been proposed in the past that light intensity might influence the distribution of cyanobacteria in the water column [21] but it was suggested that the impact of light intensity is low as compared with light quality. Here we provide an experimental proof that the light intensity plays an important role in the vertical distribution of seven *Synechococcus* spp. strains isolated from the littoral zone of Lake Constance in Germany [22]. While three PE-rich strains from 7.0 m water depth cultivated under laboratory conditions and exposed to light stress showed reduced levels of Chl *a* and photoprotective carotenoids, four PC-rich strains from 1.0 or 0.5 m water depths had either constant or increased levels of these pigments. Similarly, the steady-state level of the D1 protein from PSII reaction centre was strongly reduced during light stress in strains from 7.0 and 1.0 m depth but remained constant in strains from 0.5 m, suggesting better photoprotective or more efficient repair mechanisms.

## Materials and Methods

### Strains and growth conditions

The seven selected *Synechococcus* spp. strains examined in this study were isolated in the year 2000 from two series of tiles (samples were collected from the beginning of March to mid of April and from end of August to mid of October) placed for six weeks at different water depths (7.0, 1.0, and 0.5 m) in the littoral zone of Lake Constance (47° 41′N 09° 07′E, 476 km^2^, maximal depth 254 m), a large oligotrophic prealpine fresh water lake in Germany (details in [22]). As proposed previously [22], we refer to these strains as epibenthic (collected from submerged tiles) in order to distinguish them from pelagic (free-living), epiphytic (collected from macrophytes) or epilithic (collected from stones). Four of these strains were PC-rich (PC1-1, PC2-1, PC3-0.5, and PC4-0.5) and three were PE-rich (PE1-7, PE2-7, and PE3-7). The number after the dash indicates the water depth from which the strain was originally collected (Table 1). Since the year 2000, non-axenic cultures (co-culture with heterotrophic bacteria) of these strains were grown on solidified BG_11_ medium (with nitrate) and BG_11o_, a medium with a low concentration of combined nitrogen due to the omission of nitrate, under controlled laboratory conditions at 19°C and a white light regime of 12 h light/12 h dark at a light intensity of 8 to 10 µmol photons·m^−2^·s^−1^ as described [22]. For experiments, colonies were picked and transferred to BG_11/N_ liquid medium [23]. Liquid cultures were grown with gentle shaking under the same conditions as described above. To obtain adequate amounts of culture for stress experiments, initial 40 ml of cultures were transferred into increasing volumes of BG_11/N_ medium after approximately two to four weeks depending on growth rates of strains until a final volume of 1,000 ml was reached.

**Table 1:** Characteristics of investigated *Synechococcus* spp. strains.

Strain	Name used	Dominant pigment	Water depth [m]	Max. O_2_ evolution [µmol O_2_·h^−1^·mg^−1^]	Doubling time [days]	Average Chl *a* [µg/cell]
**BO 0014**	PE1-7	PE	7.0	269.54±44.68	∼12	4.91.10^−9^
**BO 00410**	PE2-7	PE	7.0	359.03±33.13	∼12	9.33.10^−9^
**BO 005022**	PE3-7	PE	7.0	248.25±8.32	∼16	9.58.10^−9^
**BO 00521**	PC1-1	PC	1.0	125.63±16.53	∼10	1.44.10^−8^
**BO 00545**	PC2-1	PC	1.0	333.23±3.94	∼13	1.08.10^−8^
**BO 00703**	PC3-0.5	PC	0.5	249.23±36.10	∼12	9.45.10^−8^
**BO 00715**	PC4-0.5	PC	0.5	108.14±25.58	∼6	1.70.10^−8^

Abbreviations used: Chl *a*, chlorophyll *a*; PE, phycoerythrin; PC, phycocyanin. Each data point is the average obtained from three independent experiments (± SD).

The contamination of cyanobacterial cultures with heterotrophic bacteria was visualised with help of epifluorescence microscopy after 4′,6-diamidino-2-phenylindole (DAPI) staining. For this reason 5 µl of the DAPI solution (20 mg ml^−1^) was applied to 50 µl of co-cultures and cells were incubated for 25 min at 25°C on the shaker at 450 rpm. After staining cells were collected on black Millipore Isopore membrane filters (Millipore, Schwalbach, Germany) and visualised by an Axiophot fluorescence microscope (Zeiss, Göttingen, Germany) equipped with a DAPI filter (excitation at 359 nm and emission at 461 nm, Zeiss, Germany) at 40× magnification. A red Chl fluorescence of photosynthetic cyanobacteria could be clearly distinguished from the blue fluorescence of DAPI-stained DNA of heterotrophic cultures (Figure S1).

### Oxygen evolution measurements

Oxygen evolution rates were measured at increasing light intensities to determine the light intensity at which photosynthesis of cyanobacterial strains is saturated. Therefore, 1.5 ml of cultures with 1.5·10^8^–1.8·10^8^ cells ml^−1^ were exposed to increasing light intensities of 8, 15, 22, 47, 60, 75, 97, 156, 420, 600, and 950 µmol photons·m^−2^·s^−1^ in an opaque DW2 liquid-phase oxygen electrode unit connected to a Respire 1 electrode control box (both Hansatech, King's Lynn, GB) at a constant temperature of 19°C. Light was applied by a KL 1500 LCD light source (Schott, Mainz, Germany). To avoid carbon depletion, 30 µl of a 200 mM NaHCO_3_ solution were added to the stirred culture before beginning of the measurement.

Before starting, the Servogore 120 recorder (BCC Goertz Metrawatt, Austria) was calibrated using sodium dithionite-treated double-distilled H_2_O for base line determination and oxygen-saturated double-distilled H_2_O for 100% saturation conditions. Measurements were performed with 15 min dark adaptation of cells, where the last 5 min were recorded, followed by 5 min of exposure to light intensities between 8 and 950 µmol photons·m^−2^·s^−1^ and a final 5 min measurement in the dark to estimate cellular respiration. Gross photosynthesis rate was obtained by addition of dark respiration to the amount of oxygen produced in light.

### Light stress treatment

Since cultures were non-axenic, a Chl *a* to cell number correlation was calculated to ensure that an equal amount of cells was used for each strain in light stress experiments. For this reason, series of dilutions including four dilution steps were prepared from two-week-old cultures in the growth phase. For each dilution step, the Chl *a* concentration was determined in three biological replicates with each measurement performed twice. To determine the amount of cells in the corresponding sample, cyanobacteria were counted twice independently in a semen fertility counting chamber with 0.1 mm depth (Hawksley, Lancing, GB). To exclude any effects of culture age, the experiments were repeated with one-week-old and three-week-old cultures giving comparable results (data not shown). Cultures subjected to light stress experiments were normalised to approximately 1.8·10^8^ cells ml^−1^ by dilution with BG_11/N_ medium. Nine aliquots of 110 ml of culture were transferred into sterile 250 ml tissue culture flasks and exposed to 200 µmol photons·m^−2^·s^−1^ (L 65W/25 S universal white bulbs, Osram, Munich, Germany) under gentle shaking for indicated periods of time. Three aliquots were subsequently transferred to recovery conditions at 8 to 10 µmol photons·m^−2^·s^−1^ for 1, 2 and 16 h. Photon fluency rates were measured with a quantum-radiometer (LiCOR, inc., model LI 185B, Lincoln, Nebraska, USA). Samples were stored at −20°C prior to analysis.

### Pigment analysis

Pigment analysis was performed by several independent methods. For growth measurements, Chl *a* concentration was determined by incubating 100 µl of cyanobacterial samples in 900 µl 100% methanol at 60°C for 10 min. The cell debris was separated from the pigment-containing supernatant by centrifugation at 25,000 g at room temperature. The absorption of the supernatant was measured at 665 nm and 750 nm and Chl *a* concentration was calculated based on the Lambert-Beer equation using the specific absorption coefficient for Chl *a* in 90% (v/v) methanol as described in [24]. To obtain relative Chl *a* values, Chl *a* was determined in each individual sample before and after light stress treatment. Total carotenoids were extracted in 95% (v/v) dimethyl formamide for 5 min at room temperature and collected by centrifugation for 5 min at 20,800 g. The pigment-containing supernatant was measured at 461 nm and 664 nm and total carotenoid concentration was calculated according to [25]. Relative amounts of total carotenoids were calculated from initially determined Chl *a* concentrations. For a more detailed analysis of changes in pigment composition (Chl degradation products, individual carotenoids and their degradation products), cells were frozen in liquid nitrogen, disrupted by grinding, lyophilised for two days and extracted in 100% acetone for one day at 4°C. Afterwards, UV/VIS absorption spectra were recorded from 330 to 750 nm at a spectral bandwidth of 0.5 nm and with 0.2 nm sampling interval in a Lambda 750 UV/VIS/NIR spectrometer (Perkin-Elmer, Waltham, Massachusetts, USA). Finally, pigments were determined by fitting with Gauss-Peak-Spectra as described in [26].

### Protein analysis

Total protein was extracted as previously described [27]. Routinely, total protein samples were loaded onto 15% or 17.5% Laemmli gels [28]. For western blotting, 6 µg of total protein were loaded onto the gels prior to their transfer to Hybond-P membranes (Amersham Biosciences, Uppsala, Sweden) using a semi-dry blotting system (Biometra, Göttingen, Germany). Primary antibodies against the D1 protein from the PSII reaction centre (a global α-PsbA antibody raised against a synthetic peptide mixture derived from the C-terminus of plant, algal and cyanobacterial D1 protein sequences) were purchased from AgriSera AB (Vännäs, Sweden), against B-PE (Bangiales phycoerythrin) from Rockland Immunochemicals (Gilbertsville, USA) and against PC-APC from Biogenesis (Kidlington, UK) and used in the dilutions recommended by suppliers. Blots were developed using horseradish peroxidase-coupled IgG (Sigma-Aldrich, Hamburg, Germany) followed by chemiluminescence detection (GE Healthcare, UK). Western blot signals were quantified (area and pixel values) with help of the ImageJ program [29].

### Statistical analysis

Growth curves, oxygen evolution measurements, light stress experiments and western blots were conducted as three independent replicates for each strain investigated. Determination of pigment levels (Chl *a* and total carotenoids) was also performed in three independent experiments for each strain with two replicates for each time point of the stress kinetics.

For statistical analyses, two-way ANOVA was performed with subsequent multiple pairwise (meaning e.g. all time points of the time course were compared with each other rather than comparisons of all time points versus a “reference”) comparisons via the Holm-Sidak method with adjustment of the significance level for multiple comparisons. This was done with SigmaPlot 11 (Systat Software Inc., www.systat.com).

## Results

### Growth characteristics of *Synechococcus* spp. strains

Non-axenic cultures of epibenthic *Synechococcus* spp. genotypes collected from tiles submerged at different water depths of Lake Constance in Germany [22] were cultivated under controlled laboratory conditions as described in Materials and Methods since time of their isolation in the year 2000. Four strains collected from 0.5 and 1.0 m water depths were PC-rich and therefore appeared green, while three strains collected from 7.0 m water depth were enriched in PE and appeared red (Table 1). It was proposed in the past that the presence of cyanobacteria with a certain pigmentation in different habitats of the aquatic ecosystem might be the result of acclimation or a local (endemic) adaptive radiation of these organisms [30], [31]. Furthermore, it was reported that six of these strains (PE1-7, PE2-7, PE3-7, PC1-1, PC2-1, and PC3-0.5) were closely related and belonged to Subalpine Cluster II, while strain PC4-0.5 was assigned to the *Cyanobium gracile* Cluster based on sequence comparison of the long 16S–23S internal transcribed spacer [22].

Since cyanobacterial cultures were non-axenic, both Chl *a* concentration and optical density (OD) of cells at 750 nm were measured for each strain to assay their growth rates. All cultures grew very slowly and there were clear differences in growth rates of individual strains (Figure S2). Strains PE1-7 and PC1-1 had the slowest growth rate leaving the lag phase after approximately 10 to 15 days after dilution, while strains PE2-7, PE3-7, PC2-1, and PC3-05 were slightly faster and entered the growth phase after 7 to 10 days. Strain PC4-0.5 started growth already after 3 to 5 days (Figure S2). The doubling time during exponential growth of the cultures was between 6 (strain PC4-0.5) and 16 (strain PE3-7) days (Table 1), as compared to the cyanobacterial model strain *Synechocystis* sp. PCC6803 with the culture doubling time of 6 days (not shown). Notably, strains stayed in the growth phase for approximately 30 (PE1-7, PE2-7, PE3-7 and PC5-0.5) or more than 40 (PC1-1, PC2-1, and PC3-0.5) days prior to reaching the stationary phase (Figure S2).

### Oxygen evolution at various light intensities

The consequence of photoinhibition of photosynthesis after exposure of cells to light stress is a reduction of the maximum quantum yield for oxygen evolution [32]. In order to determine the threshold for photoinhibition, *Synechococcus* spp. strains preadapted to low intensity light (8 to 10 µmol photons·m^−2^·s^−1^) were exposed to increasing light intensities and gross photosynthetic oxygen release was measured.

**Figure 1:**
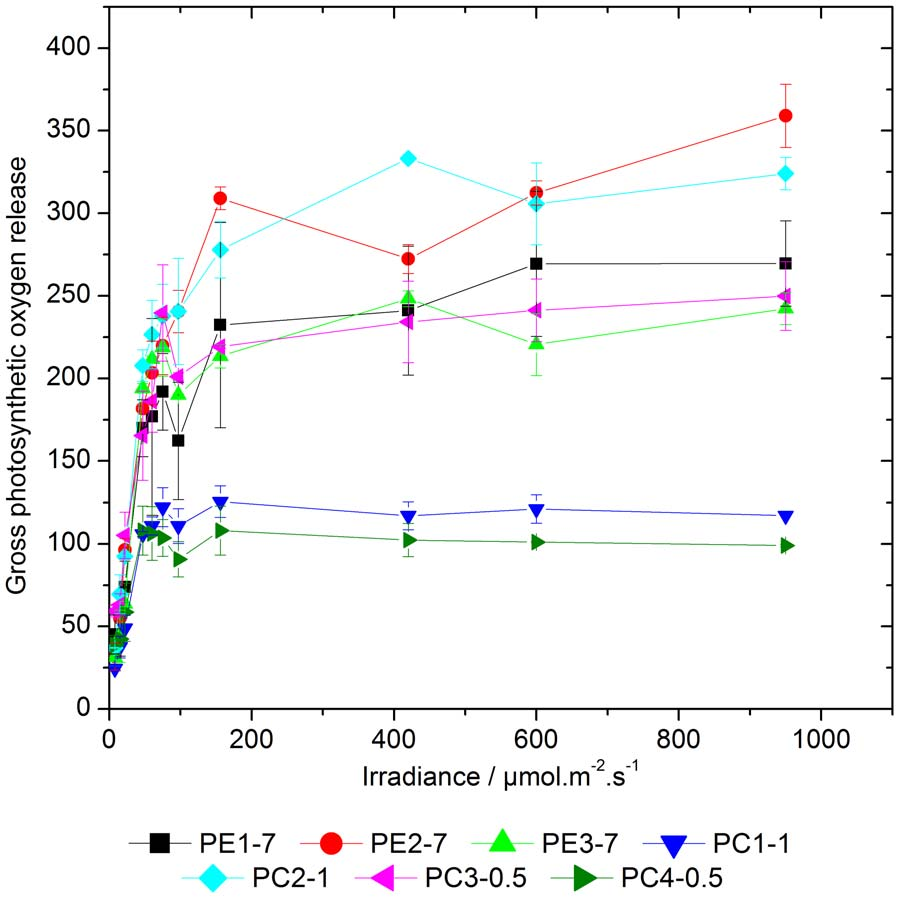
Light intensity-dependent rates of photosynthetic oxygen evolution in different *Synechococcus* spp. strains. Measurements were performed with 15 min dark adaptation, where the last 5 min were recorded, followed by 5 min exposure to light intensity of 8, 15, 22, 47, 60, 75, 97, 156, 420, 600, and 950 µmol photons·m^−2^·s^−1^ and a final 5 min measurement in the dark to estimate cellular respiration. Gross photosynthesis rate was obtained by addition of dark respiration to the amount of oxygen produced in light. A light intensity of 200 µmol photons m^−2^ s^−1^ was chosen for further light stress experiments because at this light intensity photosynthesis is saturated in all strains investigated. Error bars represent standard errors.

As shown in Figure 1, all cyanobacterial strains reached saturation of their photosynthetic capacity at approximately 100 to 150 µmol photons·m^−2^·s^−1^ and the oxygen evolution remained constant (PC1-1 and PC4-0.5) or slightly increased (PE1-7, PE2-7, PE3-7, PC2-1, and PC3-0.5) with increasing light intensities. Therefore, the light intensity of 200 µmol photons·m^−2^·s^−1^ was used in further light stress experiments (see below). This light intensity causes reversible photoinhibition in all strains analysed without being lethal for cells. Interestingly, various strains exhibited enormous differences in the maximal amount of produced oxygen. While strains PC1-1 and PC4-0.5 were capable to evolve maximally 125 and 108 µmol O_2_ h^−1^·mg^−1^ Chl *a*, respectively, strains PE1-7, PE3-7, and PC3-0.5 had a significantly higher maximal oxygen evolution rate of 269, 248 or 249 µmol O_2_ h^−1^·mg^−1^ Chl *a*, respectively (Table 1 and Figure 1). The highest oxygen evolution rate was measured for strains PE2-7 and PC2-1 and was between 359 or 333 µmol O_2_ h^−1^·mg^−1^ Chl *a*, respectively (Table 1 and Figure 1). Statistical analysis (2-way ANOVA) revealed that, looking at the whole experiment, differences in oxygen evolution were significant between most strains. Due to the noise of the measurement, less differences were significant when looking at the individual points of the curves. While no significant differences were found at the lowest irradiances, most significant differences were present at two highest light intensities applied, showing that the strains mainly differ in their maximal photosynthetic capacity.

### Responses of cyanobacterial strains to light stress at the pigment level

**Figure 2:**
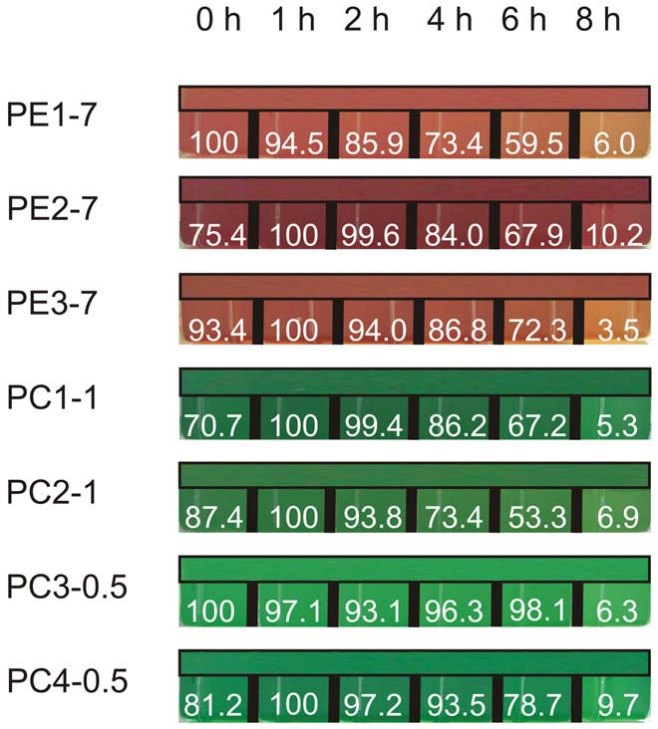
Different *Synechococcus* spp. cultures after exposure to a light intensity of 200 µmol photons·m^−2^·s^−1^ for 0 to 8 h. Bars placed above individual kinetics represent the coloration of cultures prior to light stress exposure (0 h) in order to allow better comparison. The level of pigmentation was quantified (area and pixel values) with help of the ImageJ program [29]. The maximal value was set as 100%.

**Figure 3:**
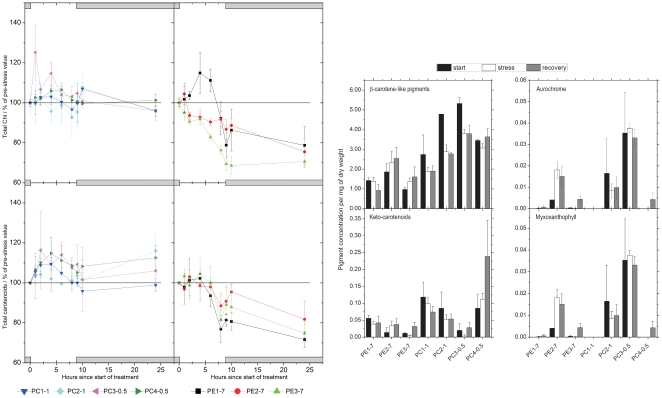
Analysis of changes in pigment contents during light stress and recovery in low light pre-adapted PC-rich (left panels) and PE-rich (right panels) *Synechococcus* spp. strains. (A) Kinetics of the amounts of total Chl *a* (upper panels) and total carotenoids (lower panels) in low light (8 to 10 µmol photons·m^−2^·s^−1^)-pre-adapted strains after 1 to 8 h of light stress exposure (200 µmol photons·m^−2^·s^−1^) and recovery phase (8 to 10 µmol photons·m^−2^·s^−1^) of 1, 2 or 16 h. The 0 time values (control) were set as 100%. Grey bars above and below graphs indicate time periods with low irradiance. (B) Detailed analysis of changes in individual pigments between the initial state, during light stress (200 µmol photons·m^−2^·s^−1^) and during recovery (8 to 10 µmol photons·m^−2^·s^−1^). Time points in each period averaged for noise reduction. Error bars represent standard errors.

**Figure 4:**
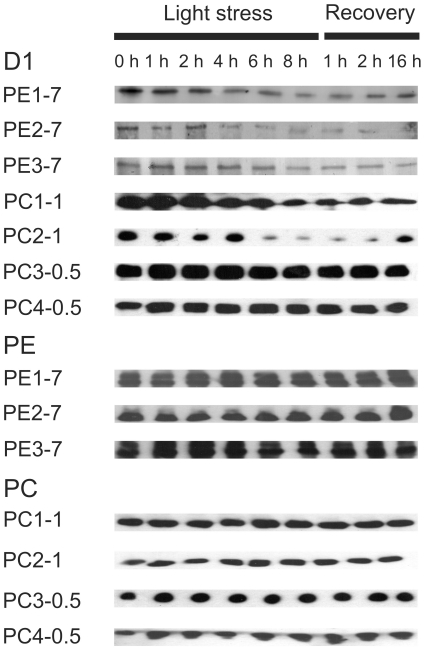
Immunoblot analysis using antibodies against the D1 protein of PSII reaction centre, PE and PC. Low light-preadapted (8 to 10 µmol photons·m^−2^·s^−1^) *Synechococcus* spp. strains were exposed to light stress (200 µmol photons·m^−2^·s^−1^) for 8 h prior to recovery at low light conditions (8 to 10 µmol photons·m^−2^·s^−1^) for 1, 2 and 16 h. Total protein was isolated and used for SDS-PAGE and immunoblotting as described in Materials and Methods. Gels were loaded on an equal protein basis (6 µg protein).

In order to assay light stress responses of cyanobacterial strains, low light-preadapted (8 to 10 µmol photons·m^−2^·s^−1^) cultures were exposed to high light conditions (200 µmol photons·m^−2^·s^−1^) followed by recovery at 8 to 10 µmol photons·m^−2^·s^−1^ prior to analysis of pigments (Figure 2 and 3) and proteins (Figure 4 and S3). Results revealed that strain PE1-7 showed a visible photobleaching already after 2 h of light stress exposure. After 4 h, all PE-rich strains and two PC-rich strains from 1.0 m water depth, but not those from 0.5 m water depth, showed a clear photobleaching of pigments (Figure 2). After 8 h of light stress exposure, all strains had a photobleached phenotype

Additionally, quantitative and qualitative analyses of relative pigment concentrations were performed in various strains exposed to light stress and during the subsequent recovery phase. The results of such analysis revealed that the relative amounts of Chl *a* measured before and after exposure to light stress conditions decreased almost linearly with the time of light stress exposure in strains PE2-7 and PE3-7 (Figure 3A, upper right panel). Statistical analysis showed that results for the strain PE3-7 significantly differed from those obtained for all PC-rich strains, while strain PE2-7 significantly differed from strains PC3-0.5, PC4-0.5, and PC2-1. In the third PE-rich strain, PE1-7, the relative amount of Chl *a* increased slightly during 4 h of light stress exposure and decreased rapidly thereafter (Figure 3A, upper right panel). Significant differences in relative amounts of Chl *a* in this strain could only be found as compared to strain PC3-0.5. No significant increase in the relative Chl *a* content was assayed during the recovery phase in all three PE-rich strains (Figure 3A, upper right panel). In contrast, in strains PC1-1, PC2-1, and PC4-0.5, relative amounts of Chl *a* remained constant during light stress and the subsequent recovery at low light conditions (Figure 3A, upper left panel). In the strain PC3-0.5, a non-significant increase in the relative Chl *a* level was measured during 4 h of light stress exposure and its level decreased thereafter to the initial value (Figure 3A, upper left panel).

Similar experiments were performed to assay total amounts of carotenoids relative to the initial Chl *a* concentration (Figure 3A, lower panels). In strains PE1-7, PE2-7, and PE3-7, the relative level of total carotenoids remained constant or slightly changed until 6 h of light stress and decreased thereafter. The level of carotenoids decreased continuously during the whole recovery phase (Figure 3A, lower right panel). In the strain PE2-7, the relative total carotenoid level did not change significantly during the light stress and the subsequent recovery phase. The PC-rich strains isolated from 1.0 m water depth showed only a slight increase (strain PC1-1) or small fluctuations (strain PC2-1) in the amount of carotenoids, while both strains collected from 0.5 m exhibited an increased level of total carotenoids relative to Chl *a* under light stress conditions and during the recovery phase (Figure 3A, lower left panel). Statistical analysis revealed that in general total amounts of carotenoids relative to the initial Chl *a* concentration in all PE-rich strains exposed to light stress and subsequent recovery conditions significantly differed from those measured for PC-rich strains.

Analysing absolute levels of individual pigments (Figure 3B), further differences between the strains could be observed. First, all PC-rich strains generally contained more Chl *a*, zeaxanthin and β-carotene-like pigments (β-carotene and β-cryptoxanthin) per dry weight than the PE-rich strains. More importantly, there were differences between individual strains. Strains PE2-7 and PC3-0.5 had more β-carotene-like pigments and zeaxanthin (these pigments have the same chromophore so that they cannot be distinguished by UV/VIS spectroscopy) and more myxoxanthophyll than other PC- and PE-rich strains (Figure 3B). Surprisingly, both strains also had the highest levels of the 5,8,5′,8′-diepoxide oxidation products of the β-carotene-like pigments ( = aurochrome in the case of β-carotene). These differences in pigmentation were constitutive, i.e. they were observed before, during and after the light stress treatment. Large differences between strains were also found for keto-carotenoids (echinenone and derivatives, Figure 3B).

### Responses of cyanobacterial strains to light stress at the protein level

We investigated changes in the steady-state level of selected proteins, such as PE, PC and the D1 protein from the PSII reaction centre in strains exposed to light stress and during recovery conditions.

The drastic differences in the D1 protein level were assayed between individual strains as demonstrated by immunoblotting. All PE-rich strains from 7 m water depth and two PC-rich strains from 1.0 m water depth showed a strong decrease in the D1 protein level during light stress treatment (Figure 4 and S3A). While the D1 protein level remained low also during the recovery phase in all PE-rich strains, and in one PC-rich strain from 1.0 m water depth (PC1-1), the second PC-rich strain PC2-1 showed an increase in the total amount of the D1 protein after 16 h of recovery. In contrast, two PC-rich strains collected from 0.5 m water depth showed only small changes in the D1 protein level under light stress but the amount of this protein slightly decreased during the recovery (Figure 4 and S3A).

In contrast to the D1 protein level, immunoblot analysis revealed no drastic changes in the phycobiliprotein levels in all strains analysed (Figure 4 and S3B), although a reduction in the general pigmentation was clearly visible in two PE-rich strains (PE1-7 and PE3-7) after 4 h of light stress exposure (compare with Figure 2).

## Discussion

### Phylogenetically closely related *Synechococcus* spp. genotypes respond differently to light stress

As previously reported for pelagic PE-rich *Synechococcus* spp., light can influence the abundance of certain cyanobacterial genotypes in co-cultures [21]. For the phylogenetically closely related cyanobacteria [22] selected for this study this might be of great importance concerning occurrence in their niche. As shown by measurement of oxygen evolution at increasing light intensities (Table 1 and Figure 1), the analysed cyanobacterial strains showed different maximal oxygen evolution rates indicating different photosystem I (PSI) to PSII ratios at similar concentrations of Chl *a*. Since the oxygen evolving complex of PSII is responsible for oxygen production and PSII was reported to be the primary target of light stress [4], one would expect that a high PSI to PSII ratio would lead to a low rate of oxygen evolution and a reduced susceptibility to light stress. Changes in the PSI to PSII ratio represent an important protective mechanism in responses to light stress in cyanobacteria [33], as well as in higher plants [34], [35]. Since strain PC4-0.5 showed a low photobleaching rate (Figure 2), the lowest oxygen evolution rate as compared to other cyanobacterial strains (Table 1 and Figure 1 and S3A) and a constant steady-state level of the D1 protein under light stress conditions (Figure 4), it can be assumed that a high PSI to PSII ratio positively influenced the response to light stress in this specific strain. Similarly to strain PC4-0.5, also strain PC1-1 showed a comparable low photobleaching rate (Figure 2) and a low maximal oxygen evolution rate (Table 1 and Figure 1).

Interestingly, the steady-state level of the D1 protein strongly decreased under light stress conditions in strains PC1-1 and PC2-1, but not in strains PC3-0.5 and PC4-0.5 (Figure 4 and S3A) suggesting a more efficient repair mechanism of photodamaged PSII in the two latter strains. The PSII repair mechanism involves a degradation of photodamaged D1 protein and its replacement by the newly synthesised functional copy [4]–[8]. Impairment of various processes that protect PSII against photoinhibition, including photorespiration, thermal dissipation of excitation energy, and the cyclic transport of electrons, decreases the rate of repair of PSII via the suppression of the D1 protein synthesis in the chloroplast [36]. This might lead to the situation where *de novo* synthesis and replacement of photodamaged D1 protein did not cope with the rate of degradation, thus leading to the reduction of its steady-state level in PC1-1 and PC2-1 strains.

The protective role of carotenoids, especially the xanthophyll zeaxanthin, has been reported in higher plants and cyanobacteria [12], [18], [37]. Therefore, an increase of total carotenoid content per cell, as well as a relative increase of zeaxanthin concentration, was expected in light stress-resistant cyanobacterial strains from 0.5 and 1.0 m water depth. The results obtained in this study are in agreement with the role of carotenoids in photoprotection because elevated levels of total carotenoids were detected in light stress-resistant PC-rich strains collected close to the water surface (Figure 3A, lower left panel). Simultaneously, the relative amounts of total carotenoids in light stress-sensitive PE-rich strains from 7.0 m water depth were reduced (Figure 3A, lower right panel), implying a low efficiency of biosynthesis of carotenoids or their enhanced degradation in these strains. Assay of the individual carotenoid composition of the different cyanobacterial strains according to [26], as well as preliminary results obtained by HPLC analysis (data not shown) demonstrated the presence of β-carotene, β-cryptoxanthin and zeaxanthin as dominant carotenoids (Figure 3B). Individual carotenoid contents normalised to dry weight showed higher amounts of protective carotenoids in PC-rich strains and the strain PE2-7 (Figure 3B), which might explain their better performance during light stress exposure. Moreover, it has been reported that the cyanobacterial pigment myxoxanthophyll plays a role in photoprotection [38]. The results of individual carotenoid analysis also revealed a strong induction of myxoxanthophyll synthesis in strains PE2-7 and PC3-0.5 as compared to other strains investigated (Figure 3B). These findings underline the important role of carotenoids in photoprotection and help to understand the physiological differences in the epibenthic cyanobacterial isolates in this study.

Based on the obtained results, one can conclude that the cyanobacterial strains analysed in this study differ in two of the photoprotective mechanisms, such as the accumulation of photoprotective carotenoids and the efficiency of the PSII repair cycle. However, it is still not clear whether other mechanisms contribute to the photoprotection observed in some strains. The influence of enzymatic radical scavenging systems, such as superoxide dismutases, catalases, and ascorbate peroxidases [39], [40] still needs to be investigated.

State transitions have also been shown to be an important protective mechanism in cyanobacteria, green algae and higher plants [41]–[43], although not much is known about the mechanism of state transition in cyanobacteria. Recent measurements of Chl fluorescence recovery after photobleaching (FRAP) favour a mobile model in which the PBS diffusion at the thylakoid membrane occurs between PSII and PSI to balance light absorption under light stress conditions [42]. In a more recent study, even reversible coupling of individual phycobiliproteins was found during light acclimation in the marine cyanobacterium *Trichodesmium erythraeum* IMS101 [10]. Such a detachment of individual phycobiliproteins under light stress conditions or their replacement by different isoforms with a lower chromophore-binding capacity might be especially important in strains unable to perform CCA (see below). In such strains, the reduction in the phycobilin content would diminish energy uptake and its transfer from PBS to reaction centres. Since in our experiments the levels of phycobiliproteins remained constant during light stress, although photobleaching of phycobilins was visible (compare Figure 2 and 4 or S3B), a detachment and/or a degradation of phycobilins would provide an explanation for the observed phenomenon. In favour of this assumption it should be noted that the total carotenoid level was constant and the relative Chl *a* content increased during 4 h and remained high up to 6 h of light stress treatment in strain PE1-7, while a visible photobleaching of the culture was observed (compare Figure 2 and 3A). Since it could be excluded that reduced amounts of Chl *a* and/or carotenoids are responsible for the decrease in general pigmentation in strains PE1-7, a change in the phycobilin to phycobiliprotein ratio can be assumed. To our knowledge, such a phenomenon has not yet been reported for cyanobacteria.

### Ecological implications

The influence of light quality on the distribution of cyanobacterial species is a well-studied field in ecology strongly connected to CCA [19], [20], [44], [45] and was regarded as a major parameter determining the vertical distribution of autotrophic plankton. However, the role of light intensity as a selective force has been shown to be of importance in marine cyanobacterial free-living *Prochlorococcus* populations [46], [47]. Indications of light as a selective force were also reported for pelagic PE-rich *Synechococcus* spp. isolates from Lake Constance [21]. In contrast, the influence of light intensity on the distribution of epibenthic cyanobacterial species in fresh waters has only been studied sporadically. The results presented in this study imply an important role of light intensity concerning selection and distribution of epibenthic cyanobacterial genotypes in the littoral zone of Lake Constance. It might be hypothesised that all cyanobacterial strains analysed here do not possess the ability to perform CCA, because they retained their different pigmentation during several years of cultivation under controlled laboratory conditions. Furthermore, our preliminary experiments revealed that cultivation of PC-rich strains in green light and PE-rich strains in orange light, the light qualities that promote CCA, did not change the pigmentation of investigated strains (not shown). One would also expect that during cultivation under low light conditions the PC-rich cyanobacterial strains would increase the amount of PE in PBS to harvest the more energetic green light instead of the orange light of the visible light spectrum. Moreover, it was reported [22] that PE- or PC-specific illumination was used to select for PC-rich (orange light) or PE-rich (green light) cyanobacteria for isolation of new genotypes. If the strains investigated here were able to perform CCA, they should have occurred under both conditions, which was not the case.

The constant pigmentation of PE- and PC-rich strains suggests that also light quality might play a role in their vertical distribution in the water column. Earlier reports showed that the spectrum of photosynthetic active radiation is dramatically narrowed with depth in Lake Constance. For the 500 nm wavelength, an irradiance of 1,600 µmol photons·m^−2^·s^−^1 was measured at the water surface and this value was reduced to 750 or 120 µmol photons·m^−2^·s^−^1 at 2 or 8 m water depth, respectively [48]. Similarly, for the 600 nm wavelength, an irradiation of 1,900 µmol photons·m^−2^·s^−1^ measured at the water surface was reduced to 700 or 50 µmol photons·m^−2^·s^−1^ at 2 or 8 m water depth, respectively. Since the ability to perform CCA can be excluded, thus it can be concluded that light intensity, and probably light quality, remain important selective forces for investigated *Synechococcus* spp. strains.

The results in this study revealed differences between strains at a light intensity of 200 µmol photons·m^−2^·s^−1^ that is regarded low as compared to a sunny day, when light intensity can easily exceed 1,600 µmol photons·m^−2^·s^−1^ at the water surface. Since Lake Constance developed meso-oligotrophic nutrient conditions [49] and light can penetrate the water column deeply, an average light intensity of 1,400, 1,250 and 1,000 µmol photons·m^−2^·s^−1^ was measured in the littoral zone at a sunny day in summer at 0.5, 1.0 and 2.0 m water depth, respectively [50]. However, one should consider that cyanobacteria in their natural habitats are preadapted to such high light intensities through a daily exposure to sunlight in summer and a slow increase of light intensity during the day, which is not the case for strains cultivated under controlled laboratory conditions. Further analysis of photoprotective mechanisms and the ability to perform CCA might provide information about evolutionary processes that lead to the distribution of different cyanobacteria in the littoral zone as it was found. Nevertheless, the effects of nutrient availability, pH, temperature, and interactions with other microorganisms remain important parameters influencing occurrence of distinct species in a niche.

## Supporting Information

Figure S1 **Image of DAPI-stained cyanobacterial cultures by epifluorescence microscopy.** Pictures were taken and analysed with help of the Magnafirev2.0 software (Optronics, Goleta, USA) using default settings with 3.2 s exposure to red and blue filters and 1.858 s exposure to green filter. Red spots, Chl fluorescence of cyanobacterial cells. Blue spots, fluorescence of DAPI-stained DNA of heterotrophic bacteria.
(TIFF)

Figure S2 **Growth curves of different cyanobacterial strains as measured by Chl** ***a*** **concentration and OD at 750 nm.** Cultures were grown in BG_11/N_ medium at a light intensity of 8 to 10 µmol photons·m^−2^·s^−1^ at 19°C with a light regime of 12 h light/12 h dark. Error bars represent standard errors (n = 3).
(TIFF)

Figure S3 **Quantification of western blot signals from** **Figure 4** **using densitometry scanning.** (A) The level of the D1 protein. (B) The level of phycobiliproteins. Error bars represent standard errors (n = 3). The maximal value in each of three replica for each strain was set as 100% and a mean value is shown.
(TIFF)
