# Supplementary figures and images for: Vertical Distribution of Epibenthic Freshwater Cyanobacterial Synechococcus spp. Strains Depends on Their Ability for Photoprotection
Source: PLoS One. 2011 May 18;6(5):e20134. doi: 10.1371/journal.pone.0020134 (PMC3097228; doi:10.1371/journal.pone.0020134)

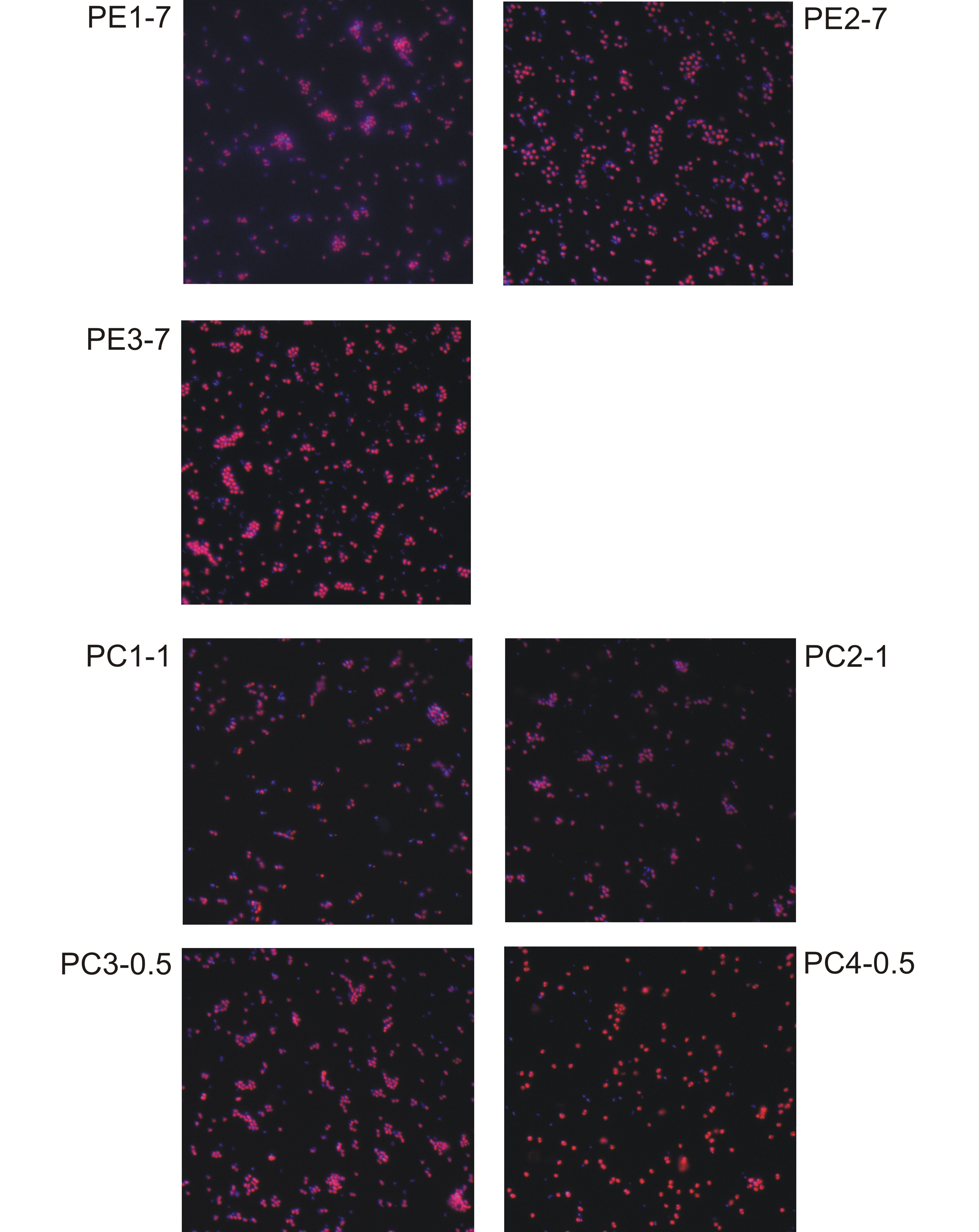

Supplement: Figure S1 — Image of DAPI-stained cyanobacterial cultures by epifluorescence microscopy. Pictures were taken and analysed with help of the Magnafirev2.0 software (Optronics, Goleta, USA) using default settings with 3.2 s exposure to red and blue filters and 1.858 s exposure to green filter. Red spots, Chl fluorescence of cyanobacterial cells. Blue spots, fluorescence of DAPI-stained DNA of heterotrophic bacteria. (TIFF) [file pone.0020134.s001.tif]

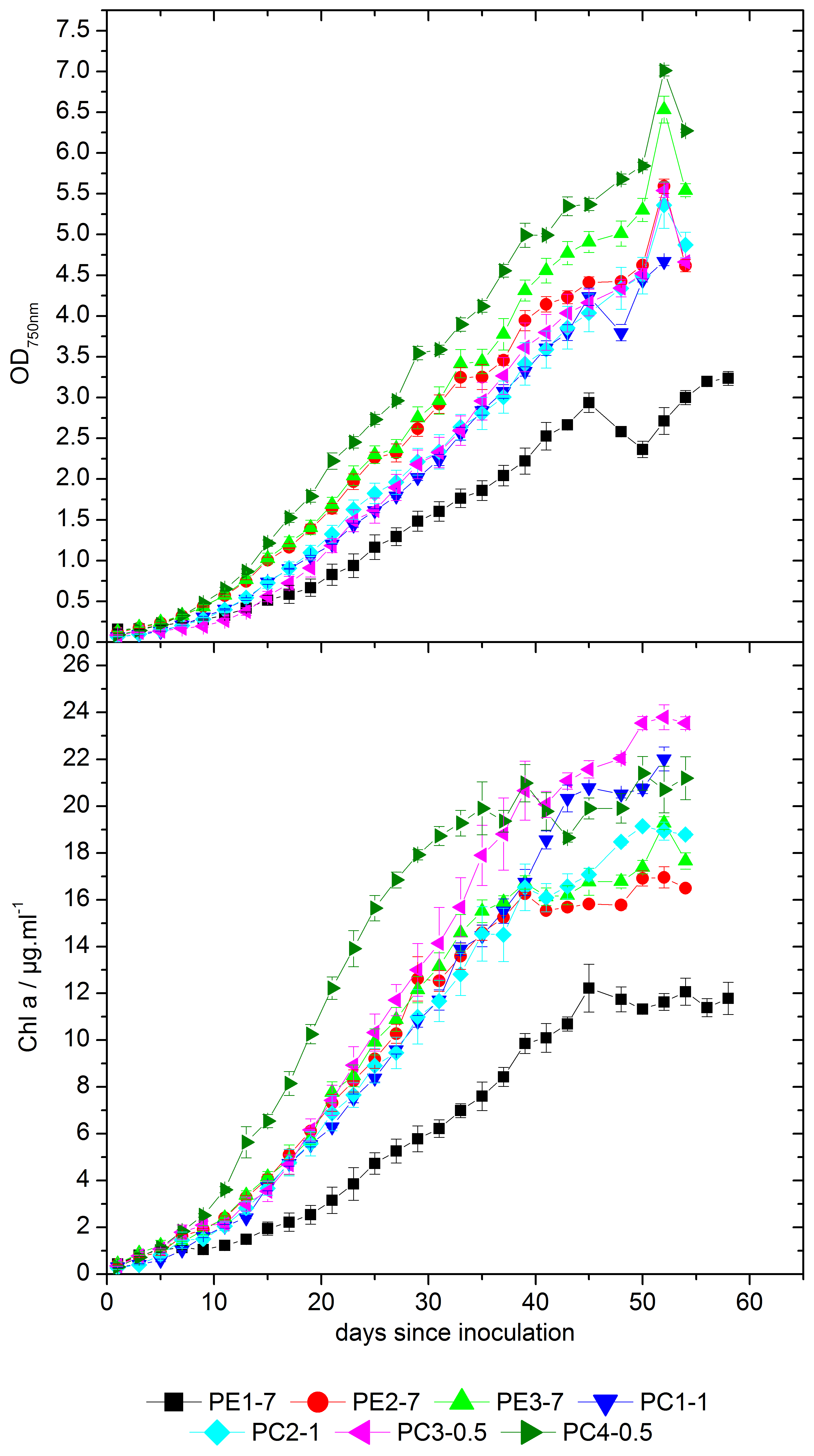

Supplement: Figure S2 — Growth curves of different cyanobacterial strains as measured by Chl a concentration and OD at 750 nm. Cultures were grown in BG11/N medium at a light intensity of 8 to 10 µmol photons·m−2·s−1 at 19°C with a light regime of 12 h light/12 h dark. Error bars represent standard errors (n = 3). (TIFF) [file pone.0020134.s002.tif]

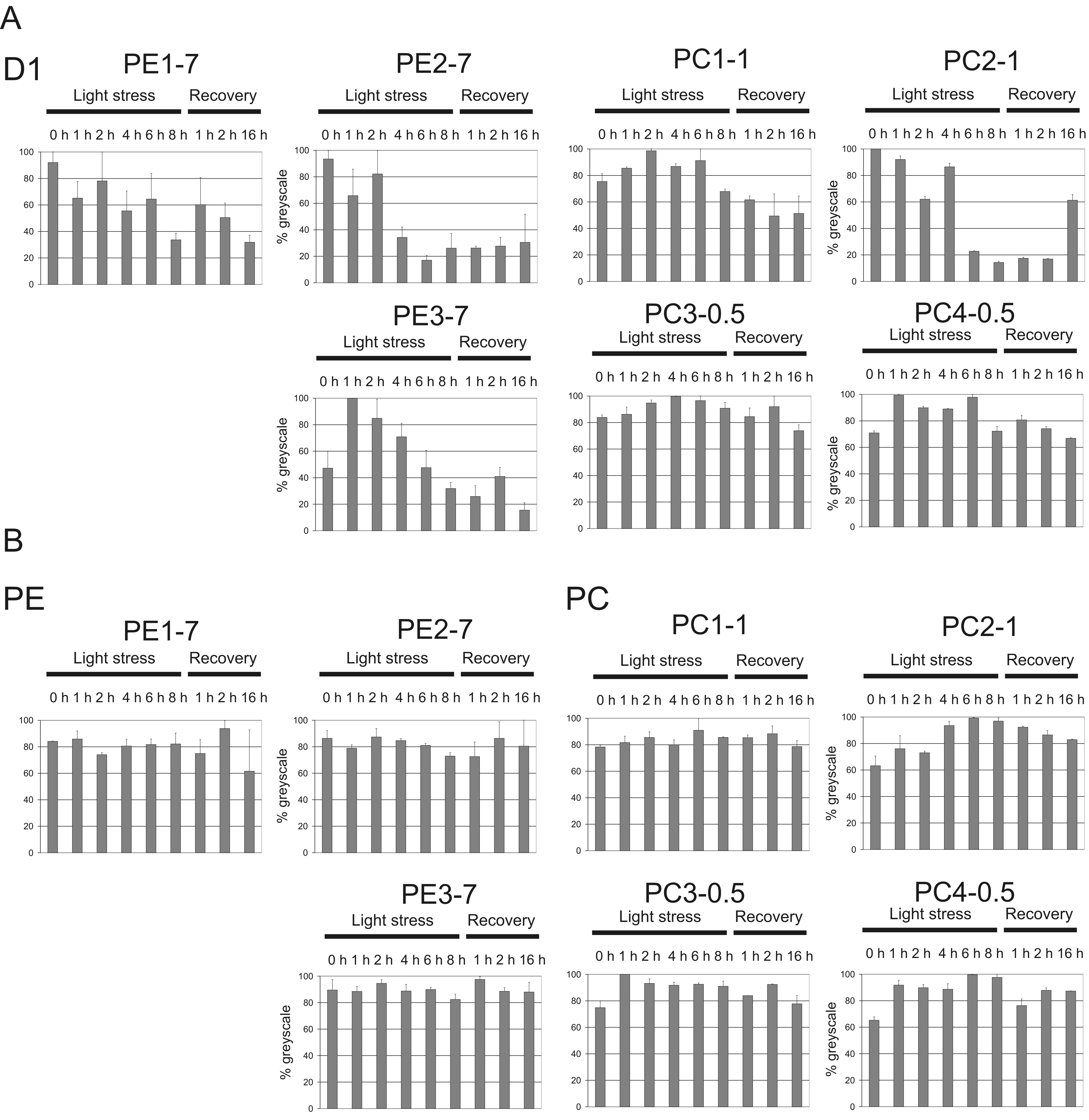

Supplement: Figure S3 — Quantification of western blot signals from Figure 4 using densitometry scanning. (A) The level of the D1 protein. (B) The level of phycobiliproteins. Error bars represent standard errors (n = 3). The maximal value in each of three replica for each strain was set as 100% and a mean value is shown. (TIFF) [file pone.0020134.s003.tif]
